# The complete chloroplast genome sequence of *Primula filchnerae* Knuth (Primulaceae), an endangered species in China

**DOI:** 10.1080/23802359.2020.1750991

**Published:** 2020-05-14

**Authors:** Yuan Lu, Pei-Liang Liu, Yi-Fang Sun, Si-Feng Li

**Affiliations:** a Xi’an Botanical Garden of Shaanxi Province, Institute of Botany of Shaanxi Province, Xi’an, China; b Shaanxi Engineering Research Centre for Conservation and Utilization of Botanical Resources, Xi’an, China; c College of Life Sciences, Northwest University, Xi’an, China; d Xianyang Vocational Technical College, Xianyang, China

**Keywords:** Chloroplast genome, *Primula filchnerae*, phylogenetic analysis

## Abstract

*Primula filchnerae* Knuth is an endangered species endemic to China. Its complete chloroplast genome was reported in this study for the first time. The whole chloroplast genome was 151, 443 base pairs in length with 131 genes, including 66 protein-coding genes, 37 tRNAs, and 4 rRNAs. In addition, the accD was inferred to be a pseudogene. Phylogenetic analysis showed a sister relationship between *P. filchnerae* and *P. sinensis* Sabine ex Lindl.

*Primula filchnerae* Knuth is endemic to central China (Hu 1990). It was evaluated as an Endangered species according to the IUCN (The International Union for Conservation of Nature) Red List Categories and Criteria (Qin et al. 2017). It was first described in 1905 (Knuth 1905), but was not discovered from the wild in the following century. Cultivated individuals were first reported from Chongqing, China, in 1996 (Hu 1996). Wild populations were re-discovered in Hubei Province in 2009 (Xie et al. 2009) and in Shaanxi Province in 2015 (Zhang et al. 2015). At present, wild *P. filchnerae* is only known from these two provinces with very limited populations.

Plant materials were sampled from an individual of *P*. *filchnerae* cultivated in Xi’an Botanical Garden, which was introduced from Tudiliang Village, Yangxian County, Shaanxi Province, China. Voucher specimens (numbered as Yuan Lu y0002) were collected from the Garden and were deposited at the Herbarium of Xi’an Botanical Garden of Shaanxi Province (Herbarium code: XBGH). Total genomic DNA was extracted from fresh leaves using the modified CTAB method (Doyle 1987). Genomic library was prepared with an insert size of 270 bp, and was sequenced on an Illumina Hi-Seq 2500 platform. About 2.07 G of raw reads were obtained and then the raw reads were filtered by the program Trimmomatic v.0.33 (Bolger et al. 2014). The filtered reads were used to assemble the chloroplast genome using the program NOVOPlasty (Dierckxsens et al. 2017). The assembled chloroplast genome was annotated by PGA (Qu et al. 2019) and Geneious v 9.0.2 (Kearse et al. 2012), followed by manual adjustments. The complete chloroplast genome sequence of *P. filchnerae* has been deposited in GenBank with the accession number MT181607.

The circular chloroplast DNA of *P. filchnerae* is 151, 443 bp in length with a typical quadripartite structure, comprising two inverted repeat (IR) regions each of 25,480 bp, a large single-copy (LSC) region of 82,720 bp and a small single-copy (SSC) region of 17,763 bp. The chloroplast genome of *P*. *filchnerae* contained 131 genes including 86 protein-coding genes, 37 tRNA genes, and 4 ribosomal RNA genes. Among these genes, 21 genes (*rpl2*, *ndhB*, *rps12*, *trnI-GAU*, *trnA-UGC, ndhA*, *trnA-UGC*, *trnL-GAU*, *rps12*, *rpl16*, *petD*, *petB*, *trnV-UAC*, *trnL-UAA*, *rpoC1*, *atpF*, *trnG-UCC*, *rps16*, *trnK-UUU*) have one intron and two genes (*pafI, clpP*) have two introns. The *accD* gene might be a pseudogene with internal stop codons. The overall GC content of *P. filchnerae* chloroplast genome is 37.2%.

We conducted phylogenetic reconstruction based on available chloroplast genome sequences of 10 species of the genus *Primula* L. and one sequence of *Cortusa matthioli* L. subsp. *pekinensis* (V. A. Richt.) Kitag. as the outgroup. GenBank accession numbers are given in Figure 1. All sequences were aligned by MAFFT v.7 (Katoh and Standley 2013). The best-fit nucleotide substitution model were determined in ModelFinder (Kalyaanamoorthy et al. 2017). Maximum likelihood (ML) analysis was performed using the program IQ-TREE (Nguyen et al. 2015) with a TVM + F+R2 model and 5000 ultrafast bootstrap replicates. The ML tree showed a sister relationship between *P*. *filchnerae* and *P*. *sinensis* Sabine ex Lindl. (Figure 1). Our results provide fundamental information for further evolutionary and phylogenetic researches of *Primula*.

**Figure 1.**
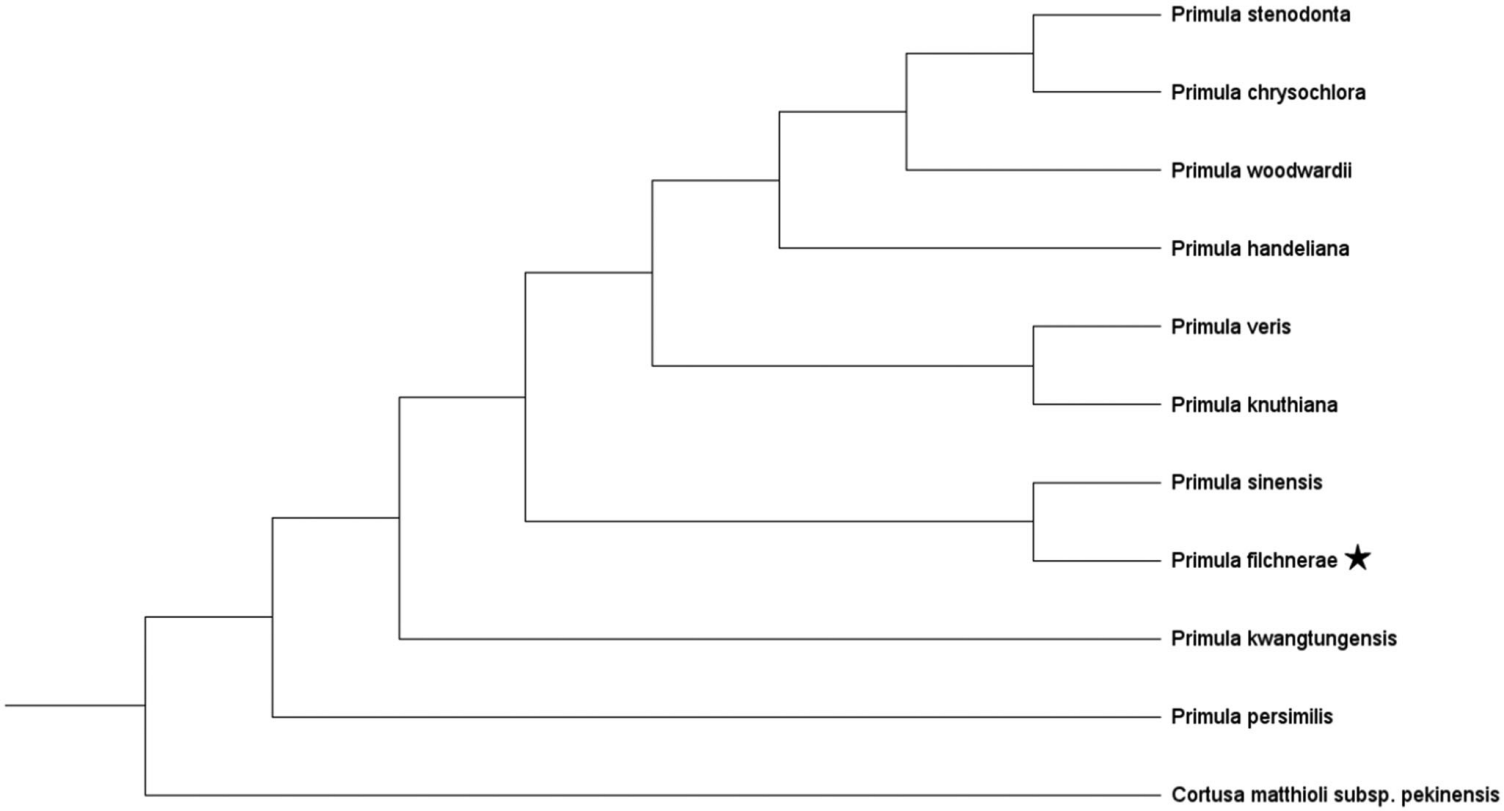
Maximum likelihood phylogenetic tree of 11 species in Primulaceae based on whole chloroplast genome data. *Primula filchnerae* is marked by a star. Bootstrap support values are shown above each branch.
